# Association of Urinary and Dietary Selenium and of Serum Selenium Species with Serum Alanine Aminotransferase in a Healthy Italian Population

**DOI:** 10.3390/antiox10101516

**Published:** 2021-09-24

**Authors:** Teresa Urbano, Tommaso Filippini, Daniela Lasagni, Tiziana De Luca, Peter Grill, Sabrina Sucato, Elisa Polledri, Guy Djeukeu Noumbi, Marcella Malavolti, Annalisa Santachiara, Thelma A. Pertinhez, Roberto Baricchi, Silvia Fustinoni, Bernhard Michalke, Marco Vinceti

**Affiliations:** 1 CREAGEN—Environmental, Genetic and Nutritional Epidemiology Research Center, Department of Biomedical, Metabolic and Neural Sciences, University of Modena and Reggio Emilia, 41125 Modena, Italy; teresa.urbano@unimore.it (T.U.); tommaso.filippini@unimore.it (T.F.); gdjeukeu@yahoo.fr (G.D.N.); marcella.malavolti@unimore.it (M.M.); 2 Transfusion Medicine Unit, Azienda USL-IRCCS of Reggio Emilia, 42123 Reggio Emilia, Italy; Daniela.Lasagni@ausl.re.it (D.L.); Tiziana.DeLuca@ausl.re.it (T.D.L.); thelma.deaguiarpertinhez@unipr.it (T.A.P.); roberto.baricchi@ausl.re.it (R.B.); 3 Research Unit Analytical BioGeoChemistry, German Research Center for Environmental Health, Helmholtz Center Munich, 85764 Neuherberg, Germany; grill@helmholtz-muenchen.de (P.G.); bernhard.michalke@helmholtz-muenchen.de (B.M.); 4 Department of Clinical Sciences and Community Health, University of Milan, 20122 Milan, Italy; sabrina.sucato@unimi.it (S.S.); elisa.polledri@unimi.it (E.P.); silvia.fustinoni@unimi.it (S.F.); 5 AVIS Provinciale, 42013 Reggio Emilia, Italy; annalisa.santachiara@avisre.it; 6 Department of Medicine and Surgery, University of Parma, 43125 Parma, Italy; 7 IRCCS Ca’ Granda Foundation Maggiore Policlinico Hospital, 20122 Milan, Italy; 8 Department of Epidemiology, Boston University School of Public Health, Boston, MA 02118, USA

**Keywords:** selenium, selenium species, exposure, alanine aminotransferase, non-alcoholic fatty liver disease, epidemiology

## Abstract

The trace element selenium is of considerable interest due to its toxic and nutritional properties, which markedly differ according to the dose and the chemical form. It has been shown that excess selenium intake increases the risk of type 2 diabetes and, possibly, other metabolic diseases like hyperlipidemia and non-alcoholic fatty liver disease (NAFLD). For the latter, however, epidemiologic evidence is still limited. We carried out a cross-sectional study recruiting 137 healthy blood donors living in Northern Italy and assessed their exposure to selenium through different methods and measuring serum selenium species. We performed linear and spline regression analyses to assess the relation of selenium and its forms with serum alanine aminotransferase (ALT) levels, a marker of NAFLD. Urinary selenium levels were positively and somewhat linearly correlated with ALT (beta regression coefficient (β) 0.11). Conversely, the association of dietary selenium intake with ALT was positive up to 100 µg/day and null above that amount (β 0.03). Total serum selenium was inversely associated with ALT up to 120 µg/L, and slightly positive above that amount. Concerning the different serum selenium species, ALT positively correlated with two organic forms, selenocysteine (β 0.27) and glutathione peroxidase-bound selenium (β 0.09), showed a U-shaped relation with the inorganic tetravalent form, selenite, and an inverse association with human serum albumin-bound selenium (β −0.56). Our results suggest that overall exposure to selenium, and more specifically to some of its chemical forms, is positively associated with ALT, even at levels so far generally considered to be safe. Our findings add to the evidence suggesting that low-dose selenium overexposure is associated with NAFLD.

## 1. Introduction

The human health effects of the trace element selenium continue to command attention in biomedical research, with particular reference to the involvement of selenium in chronic disease etiology due to its nutritional and toxicological properties [1]. Exposure to selenium mainly occurs through diet. More precisely, it is particularly abundant in offal, fish and seafood, cereals, and dairy products [2,3]. Beyond occupational environments, other routes of exposure include drinking water, soil as well as cigarette smoking, and even motorized traffic [3,4,5]. Individual dietary selenium intake ranges from 3 to 7000 µg/day across the world, with the highest levels in selected seleniferous areas of countries such as China, India, and Venezuela, intermediate levels in the US, and lower ones in European countries [2,6].

Selenium is a trace element of both toxicological and nutritional interest [1], with an uncertain but narrow, safe range of intake [6]. It is also well recognized that the health effects and biological properties of selenium, including its nutritional and toxicological effects, considerably differ according to its chemical forms, whether inorganic or organic [7,8,9,10]. To be stable, selenium needs to form compounds. This is why it does not generally occur in its elemental state, except in relation to soil [11]. Its most common inorganic forms are selenides, selenite (SeO_3_^−2^, Se [+4]) and selenate (SeO_4_^−2^, Se [+6]). The last two species are water-soluble and can be frequently found in water. Conversely, organic selenium forms include a wide range of species, including selenides, selenium amino acids, selenium-containing proteins, and selenoproteins [9,11,12]. Selenium may also be found to be bound to human serum albumin (Se-HSA), still not well defined as to its organic or inorganic nature. Both organic and inorganic selenium species are involved in oxidoreduction reaction regulation and pathways [13,14,15,16]. Selenium bound to cysteine (Se-Cys) is a component of the so-called selenoproteins, the most extensively studied of which are glutathione peroxidase-bound selenium (Se-GPX), thioredoxin reductase-bound selenium (Se-TXNRD), and selenoprotein P (SELENOP) [17,18,19].

Concerning the specific health effects of selenium exposure, this metalloid was first hypothesized to be carcinogenic, however, it has then been proposed as an anticancer agent [20]. Eventually, large randomized trials have indicated that no beneficial effect on cancer risk is owed to this element, while it may increase the risk of advanced prostate cancer and non-melanoma skin cancer [20,21]. These randomized controlled trials have also consistently documented an excess risk of diabetes as a side effect of selenium supplementation, which has raised considerable concern [22,23]. In addition, it has been suggested that a main adverse effect of selenium overexposure is hepatotoxicity [24,25,26,27]. This has long been recognized among the side effects of chronic selenosis in individuals naturally exposed to high levels of environmental selenium [28], in line with observations from laboratory animals alanine aminotransferase (ALT) levels [29].

More recently, however, it has been suggested that even low-dose selenium exposure may induce hepatotoxicity, as reflected by increased levels of ALT, a known biomarker of liver cell injury [30]. ALT circulating levels are generally considered to be in the normal range of 29–33 and 19–25 international units per liter (IU/L) for men and women, respectively [31]. High levels of ALT correlate with the severity of liver damage and represent a diagnostic biomarker of non-alcoholic fatty liver disease (NAFLD) [32], a serious condition with a large and increasing prevalence worldwide [33].

In a healthy Italian population, we investigated if circulating ALT levels could be associated with selenium exposure, as assessed through the evaluation of dietary intake, serum levels of selenium and selenium species, and urinary selenium concentrations. This was the first study, as far as we know, to assess the association of this enzyme with the different chemical forms of selenium in humans.

## 2. Materials and Methods

### 2.1. Population of the Study

Within a cross-sectional study, we recruited a consecutive series of blood donors from ‘Casa del Dono’ within the Transfusion Medicine Center of AUSL-IRCCS of Reggio Emilia, Northern Italy, in the period April 2017–April 2019. We enrolled in the study subjects who were residents in the province of Reggio Emilia, aged 30–60, a non-smoker, and affected by no known acute or chronic disease. Of the 148 subjects who accepted to participate, four did not provide informed consent and eventually withdrew from the study, while we excluded seven subjects because the cotinine levels were entirely inconsistent (>100 µg/L) with the self-reported non-smoking status [34].

Therefore, the final study population encompassed 137 people, who provided blood and urine samples. In addition, they provided detailed data about anamnestic and residential history as well as lifestyle habits. Finally, they yielded written consent to participate in the study, as required by the Reggio Emilia Ethics Committee (approval no. 0022799/2016). Moreover, we assessed participants’ diet by administering a semi-quantitative food frequency questionnaire (FFQ) specifically developed in the project ‘European Prospective Investigation into Cancer Nutrition’ (EPIC) for the Central-Northern Italy population. This validated EPIC-FFQ allows the assessment of consumption patterns in relation to 188 food items [35,36]. We computed the related intake of nutrients by using a specific food chemical composition database and an ad hoc developed software [37,38].

### 2.2. Laboratory Determinations

To determine circulating selenium, selenium species, and ALT concentrations, we collected fasting blood samples during the morning. The serum samples were separated from whole blood through centrifugation (room temperature, 4000 rpm for 10 min). We stored samples at −20 °C until analysis. We assessed ALT concentrations through automated laboratory procedures using enzymatic colorimetric assay, while urinary cotinine levels were measured by liquid chromatography coupled with tandem mass spectrometry (LC-MS/MS), according to a methodology we previously described [34].

For selenium, we collected urine samples in polypropylene tubes and serum samples using vacutainer tubes, which we then centrifuged to obtain 1 mL of serum aliquot, subsequently stored at −20 °C until analysis. Then, urine samples were thawed at room temperature for 2 h. To dissolve the sediment for the analysis, samples were mixed and heated for 30 min at 37 °C. For each sample, we transferred into a polyethylene tube an aliquot of 600 µL, and we added to an aqueous solution of nitric acid 0.05% *v*/*v* prepared by diluting ultrapure nitric acid (69% TraceSelect, Fluka, France), containing 7.5 µg/L of Scandium-45 (45Sc), Yttrium-89 (89Y) and Indium-111 (111In) (Inorganic Ventures, Inc., Lakewood, NJ, USA). Ultrapure water (conductivity 0.056 µS/cm) (Milli-Q^®^, Merck, Darmstadt, Germany) was used to prepare all solutions. We analyzed samples using inductively coupled plasma-mass spectrometry (ICP-MS) (X Series II, Thermo Electron Corporation, Rodano, Italy). The instrument was operated in collision cell mode (CCT-Ked), with 3.7 mL/minutes of helium used to reduce interference. For each sample, three replicates were run. The calibration curve was in the range of 0.2–70 µg/L and the calibration solutions were obtained by diluting a selenous acid standard solution containing selenium at 1 mg/mL (BDH, VWR International, Milan) and an aqueous solution of nitric acid 0.05% *v*/*v* in the presence of internal standards. The calibration curve was found to be linear (correlation coefficient ≥ 0.999). The limits of quantification (LOQs) amounted to 1.2 µg/L. We assessed internal quality assurance with two quality controls (QCs) for metals in urine: Lyphocheck Urine Metals Control, Level-1 (Bio-Rad Laboratories, Anaheim, CA, USA) and Seronorm^®^ Level-1 (Sero AS, Billingstad, Norway). QC accuracy and precision ranged between 90–110% and 7–11%, respectively.

Concerning serum selenium and selenium species levels, we used the following methodology. We determined total selenium and the selenium species in serum samples, namely selenite, selenate, selenocysteine (Se-Cys), selenomethionine (Se-Met), selenoprotein P (SELENOP), glutathione peroxidase-bound selenium (Se-GPX), thioredoxin reductase-bound selenium (Se-TXNRD), and human serum albumin-bound selenium (Se-HSA) by using high-pressure liquid chromatography (HPLC) along with inductively coupled plasma—dynamic reaction cell—mass spectrometry (ICP-DRC-MS), according to already established methodologies developed in our laboratory [39,40]. We used supra pure grade chemicals and reagents throughout. Selenite and selenate, Se-Cys, Se-Met, Se-GPX (EC 232-749-6), Se-TXNRD (EC 1.8.1.9.), Se-HSA and TRIS buffer were ordered from Sigma-Aldrich, Deisenhofen, Germany. SELENOP standard was prepared from serum using affinity chromatography [41], as described in detail in Vinceti et al. [42]. Certified selenium stock standards (1000 mg/L) were purchased from CPI (Santa Rosa, CA, USA). Ammonium acetate and acetic acid were from Merck, Darmstadt, Germany, whilst Arliq and methane (99.999% purity) were purchased from Air Liquide, Gröbenzell, Germany. Selenium species stock solutions were prepared at a concentration of 1000 mg Se/L by dissolving them in Milli-Q water (18.2 MΩ cm, Milli-Q system, Millipore, Bedford, MA, USA). Similarly, HSA was prepared accordingly at a concentration of 1000 mg/L. Working standards of Se-species were prepared daily from their stock standard solutions by appropriate dilution with Milli-Q H2O. We performed strong anion exchange (SAX) separation of Se-species, based on established methodology (see details in [42,43]). A Knauer 1100 Smartline inert Series gradient HPLC system served as the eluent delivery system. For species separation, an anion exchange column AS-11 (250 × 2 mm I.D.) from Thermo-Dionex (Idstein, Germany) was installed. The sample volume was 100 µL. The mobile phases were eluent A: 10 mM Tris-HAc buffer, pH 8.0; and eluent B: A + 500 mM ammonium acetate, pH 8.0, using gradient elution details described elsewhere [44]. The flow rate was set to 0.90 mL/min. Experimental settings after optimization for analysis with ICP-DRC-MS (Perkin Elmer NexIon) were as follows: radio frequency power: 1300 W; plasma gas flow: 16 L Ar/min; auxiliary gas flow: 1.05 L Ar/min; nebulizer gas flow: 0.98 L Ar/min, daily optimized; dwell time: 300 ms; ions monitored: 78Se, 80Se; DRC reaction gas: CH4 Reaction at 0.58 mL/min; DRC rejection parameter q: 0.6. Total selenium was measured with ICP-sf-MS (ELEMENT II, Thermo Scientific). The experimental settings were as follows: radio frequency power: 1260 W; plasma gas flow: 16 L Ar/min; auxiliary gas flow: 0.85 L Ar/min; nebulizer gas flow: 1.085 L Ar/min, daily optimized; ions monitored: 77Se and 78Se, both in high-resolution mode.

### 2.3. Statistical Analysis

We assessed the distribution of total selenium and individual selenium species concentrations, dietary selenium intake, and serum ALT levels in the study participants to identify extreme, implausible values. We found a subject with an extremely low serum selenium level, 22.00 µg/L, and six more with unusually high levels of Se-TXNRD (17.55 µg/L), Se-Met (30.35 µg/L), Se-Cys (16.36 µg/L), selenite (121.89 µg/L), selenate (66.44 µg/L) and Se-HSA (26.14 µg/L). These values (both the lowest and the highest) were considered as outliers. Therefore, we winsorized at the 1st or 99th percentile to minimize the influence of outliers in our data.

We evaluated the association of urinary and dietary selenium concentrations, total serum selenium levels, organic, inorganic species, and Se-HSA with ALT, by using crude and multivariable linear regression and spline regression analyses. In the analysis, we adjusted for the following potential confounders: sex, as a discrete variable, and age, body mass index (BMI), cotinine levels, and alcohol intake as continuous ones. We used a restricted cubic spline regression model for the dose-response analysis by using three knots at fixed percentiles (namely 10th, 50th, and 90th). For all effect estimates, we assessed statistical imprecision by computing their 95% confidence interval (CI). We used the routines ‘mkspline’, ‘regress’, ‘xbcrsplinei’, and ‘winsor’ from Stata 17.0 (Stata Corp., College Station, TX, USA, 2021) for all data analyses.

## 3. Results

Our population was eventually composed of 137 non-smoking healthy subjects, 62 men and 75 women. The main characteristics of study participants are summarized in Table 1. Mean urinary selenium concentrations, dietary selenium intake, and total serum selenium levels in the overall study population and for each of the two sexes are also reported (Table 1). Table 2 and Table 3 show the interquartile range (IQR) of ALT and the selenium biomarkers assessed in our study, divided by sex. Median ALT concentration amounted to 27 µg/L, (IQR: 22–35 µg/L), higher in men than in women. Median levels of dietary selenium intake, urinary selenium concentration, and total serum selenium contents were 78.7 µg/day (IQR: 62.6–101.5 µg/die), 22.0 µg/L (IQR: 14.6–37.2 µg/L) and 116.5 µg/L (IQR: 106–128 µg/L), respectively, with higher values in men than in women. The median and IQR values of the various organic and inorganic selenium species are reported in Table 3. When assessing the association between the different selenium biomarkers used in the study participants, we found a positive association between dietary intake and urinary selenium concentrations, especially above 100 µg of daily selenium intake [45]. The association between total serum and urinary selenium concentration was negative and almost linear, while that with dietary intake was U-shaped, in that it was negative until 90 µg/day and then slightly positive (Figure S1).

**Figure 1:**
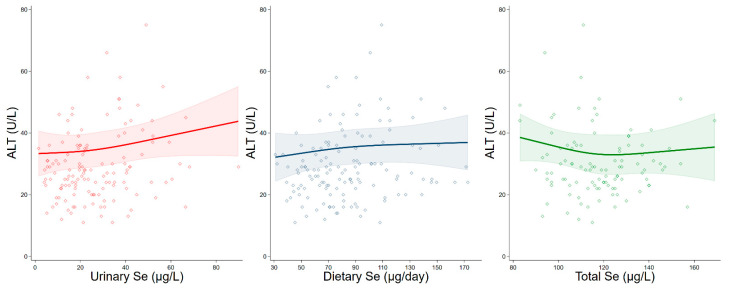
Spline regression analysis of urinary, dietary, and total serum selenium (Se) levels, versus ALT. The solid line represents the multivariable analysis (adjusted by age, sex, body mass index, cotinine levels, and intake of alcohol) with upper and lower confidence interval limits.

Using the linear regression model, we found a positive association of dietary selenium and particularly of urinary selenium concentrations with ALT, while total serum selenium concentrations were weakly and negatively associated with enzyme levels (Table 4). In spline regression analysis, we observed an almost linear positive association between urinary selenium excretion levels, especially above 30 µg/L, and ALT. In the meantime, a slightly positive association under 100 µg/L, and then an almost null one above that value, emerged for dietary selenium intake. Concerning the association with total serum selenium, spline regression analysis showed a negative association with ALT at a dose of up to 120 µg/L, then the curve inverted its direction becoming slightly positive (Figure 1).

Concerning the single selenium species and their categories, both organic and inorganic selenium overall showed no association with ALT in linear regression analysis. Conversely, such association was positive with three organic compounds, namely Se-GPX, selenomethionine (Se-Met), and Se-Cys. Little evidence of an association between ALT and the other organic (SELENOP, Se-TXNRD) and inorganic (selenite and selenate) selenium forms emerged (Table 4).

**Figure 2:**
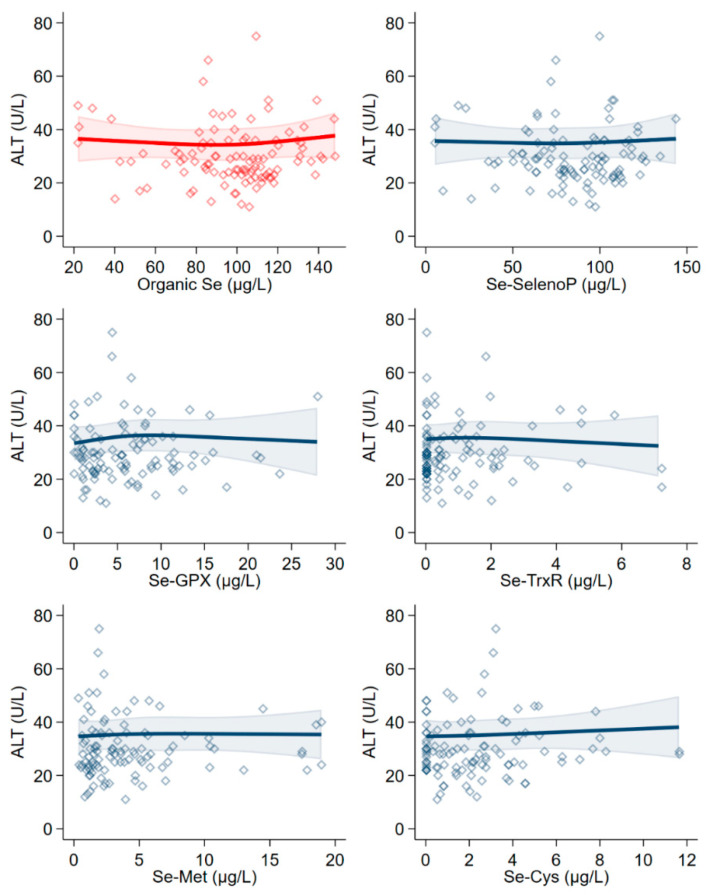
Spline regression analysis of total organic serum selenium (Se) levels and organic species, versus ALT. The solid line represents the multivariable analysis (adjusted by age, sex, body mass index, cotinine levels, and intake of alcohol) with upper and lower confidence interval limits.

In spline regression analysis, ALT showed almost no association with SELENOP and Se-TXNRD, while a slightly positive relation emerged with Se-Cys. The relation with Se-GPX showed an inverted U-shape: as such, it was firstly positive and then it became negative above a concentration of 10 µg/L (Figure 2).

**Figure 3:**
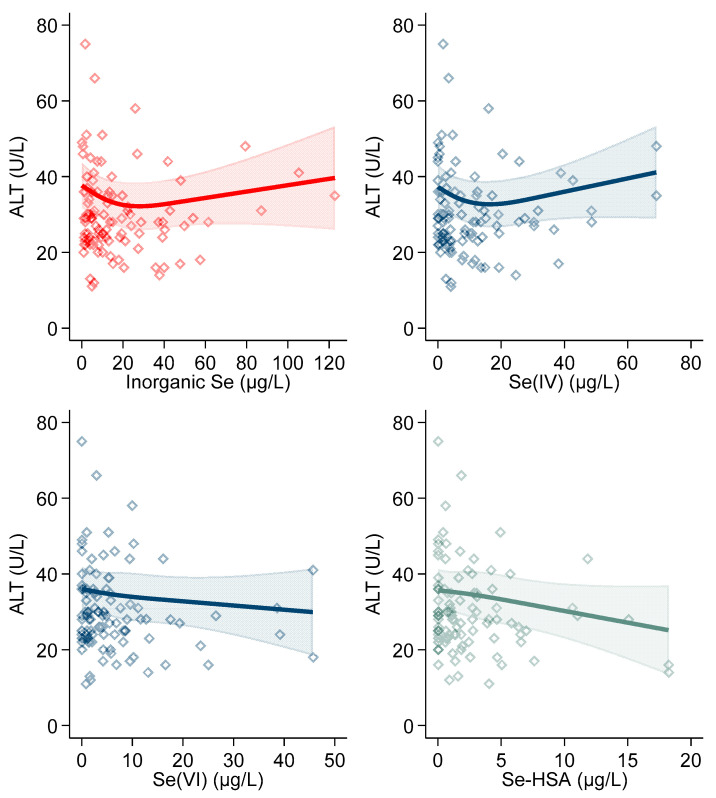
Spline regression analysis of inorganic serum selenium (Se) levels and human serum albumin-bound selenium (Se-HSA), versus ALT. The solid line represents the multivariable analysis (adjusted by age, sex, body mass index, cotinine levels, and intake of alcohol) with upper and lower confidence interval limits.

Overall, organic selenium showed a weak negative relation with ALT up to 100 µg/L, the association becoming slightly positive beyond that value. As regards inorganic selenium, this showed a negative association with ALT up to 20 µg/L, the association then becoming positive and almost linearly increased up to its maximum levels (Table 4, Figure 2 and Figure 3). A similar pattern was exhibited by tetravalent inorganic selenium (selenite), while the association of the hexavalent form selenite with ALT was negative and almost linear (Figure 3).

With reference to the Se-HSA, we observed a strong negative association with ALT in both linear regression analysis (Table 4) and spline analysis (Figure 3).

When we stratified the analysis by sex, we found a few relevant differences between men and women. In men, ALT was positively associated with urinary selenium concentrations and dietary selenium intake in the linear regression analysis, while a negative association emerged for total serum selenium. On the other hand, such associations were almost null and statistically unstable for women. As far as the various selenium species are concerned, we observed slightly negative and statistically unstable associations in men between ALT and both overall organic and inorganic selenium. ALT showed a positive association with Se-GPX and Se-Cys and a negative one with the other organic and inorganic species as well as Se-HSA. A null association was observed for selenite. In women, many of the associations were opposite to those found in men: there was a strong positive association with Se-GPX, Se-Met, and Se-Cys, and a strong negative association with Se-TXNRD. As regards the inorganic species, ALT was positively associated with total inorganic selenium. This was also true for the two individual inorganic species, namely selenate and, more strongly, selenite. The association between Se-HSA and ALT was negative and rather strong (Table S1).

Spline regression analysis confirmed the occurrence of sex-specific differences. In men, there was a strong positive association of both urinary selenium and dietary selenium intake with ALT, while overall serum selenium was negatively associated up to 120 µg/L, and positively associated at higher levels (Figure S2). In women, the association between urinary selenium biomarkers and ALT became slightly positive above 40 µg/L, while it was almost null with dietary selenium. For total serum selenium, the association with ALT was slight and negative below 120 µg/L, and positive above that value (Figure S3). Concerning overall organic selenium and SELENOP, the relation with ALT was U-shaped in men. Accordingly, it was negative under a concentration of 90 µg/L, then it flattened and became positive above that value. The remaining single organic species were generally not associated with ALT (Figure S4). Conversely, in women, the association with ALT was null for overall organic species, negative above 60 µg/L for SELENOP, positive for Se-GPX up to 7 µg/L, and then slightly negative, positive for Se-TXNRD and Se-Met, and slightly positive for Se-Cys (Figure S5). Concerning the inorganic species, selenite showed a U-shaped association with ALT, which was inverse below 20 µg/L and direct above such dose in men, while it was overall slightly direct in women. Conversely, the association with selenate was negative and almost null in men and women, respectively (Figures S6 and S7). In both sexes, Se-HSA was negatively associated with ALT, with an almost linear pattern (Figures S6 and S7).

## 4. Discussion

Interest in the role in human health of the trace element selenium has greatly risen during the last decades. Although some points have been convincingly elucidated, many controversial issues have not yet been resolved [17]. Some recent trials, particularly SELECT, have clarified that selenium does not protect against cancer risk [46] and increases site-specific cancer risk [47,48,49]. Nowadays, therefore, the main interest in selenium concerns the adverse effect of overexposure on the risk of type 2 diabetes [23,50] and related metabolic alterations, namely dyslipidemia and insulin resistance, hypertension, and specifically non-alcoholic fatty liver disease [1]. In particular, the latter outcome is generating strong interest, from the long-term identified chronic hepatotoxic effects of selenosis [24] to more recent reports documenting a positive association between selenium and ALT. One study from a seleniferous US area found a positive association between dietary selenium intake and ALT [51], while another recent study in a comparable area of India has observed no association of blood, hair, and nail selenium contents with ALT [28]. A recent study has found evidence from NHANES about a positive association between circulating levels of selenium and ALT as a marker of NAFLD [52].

In this study, we focused on the association between selenium exposure, assessed through three different indicators and specific circulating selenium chemical forms, and circulating ALT levels in a population characterized by the ‘average’ background intake of selenium characterizing most Western countries. Both urinary and blood selenium levels have been validated as suitable indicators of selenium exposure, while the direct estimate of dietary selenium intake is also of value but exposed to potential misclassification bias [53]. However, even total serum selenium content does not necessarily reflect the long-term intake of the element and, even more so, the levels of the individual selenium species [1,7]. In our study population, dietary selenium intake was positively associated with urinary selenium concentrations, especially above 100 µg of daily selenium intake (Figure S1). Conversely, the relation between total serum selenium and urinary selenium concentration was negative and almost linear, while that with dietary intake showed a sort of U-shaped association, which was negative until 90 µg/day and then slightly positive (Figure S1).

We observed a positive association between selenium exposure, assessed through the evaluation of its dietary intake and through urinary concentrations, and ALT, which may indicate a slight, subclinical hepatotoxic effect of selenium even at relatively low amounts of exposure. Such potential effect could not be linked to selenium exposure when assessed through its overall serum levels, while it was present with reference to two organic species in serum, Se-Cys and Se-GPX, and the inorganic form selenite at its highest levels. These observations are of interest since Se-Cys is a physiological selenoprotein but may also have adverse effects. However, the latter is still not well understood [54], while the inorganic form selenite is one of the most toxic forms of the element [42].

In assessing the associations mentioned earlier, two potential limitations should be taken into account. One is some statistical imprecision in the effect estimates, due to the small size of the study population, which was not vast enough to yield statistically stable estimates, particularly in subgroup analyses. The second limitation is inherent in non-experimental studies, i.e., the possibility that the associations we found were not causal, but rather confounded by unmeasured dietary and non-dietary factors. The only source of evidence that can be free from such bias stems from experimental human studies i.e., randomized controlled trials. Unfortunately, only one study has included ALT as an outcome of interest so far, and it did not report on the effects of selenium supplementation on enzymatic levels as a continuous variable [55].

These results are in keeping with two US reports. The first was carried out in a seleniferous area of South Dakota and identified a positive association between selenium dietary intake and ALT [51]. The second study reported a positive association in the National Health and Nutrition Examination Survey (NHANES) between serum selenium levels and ALT above 130 µg/L, thus suggesting an adverse effect of overexposure to this metalloid on the risk of NAFLD [52]. A key finding of our study is that the relation between serum selenium and ALT was not linear but U-shaped, with a turning point around 120 µg/L. This indicates that a relationship may only arise above that cut-point, which can be viewed as an approximate threshold. Such amount corresponds to a dietary selenium intake of around 80 µg/day [56], the intake found to be associated with an excess risk of type 2 diabetes in a recent prospective cohort study [57] as well as in a meta-analysis of observational studies [50].

To our knowledge, no studies have so far assessed the impact of different selenium species on ALT levels. Previous observational studies focused on circulating selenium levels or SELENOP concentrations in patients affected by NAFLD or non-alcoholic steatohepatitis (NASH) [58,59,60,61,62]. In our investigation, we did not find evidence of an association between SELENOP (as assessed using SELENOP-bound selenium) and ALT, while we found an association with other organic and inorganic selenium species. Replacement of methionine and cysteine with Se-Met and Se-Cys is known to lead to altered protein function and structure [63,64,65,66]. Moreover, the toxic effects of selenite encompass a wide range of deleterious effects [67,68,69,70] that can also be related to hepatotoxicity [27,71].

Overall, our key finding is that a positive and therefore potentially adverse association between selenium and ALT can be found in a moderately selenium-exposed Italian population. This relation appears to be non-linear with a threshold approximately just above the upper cut-point of the recommended dietary intake. The positive association we observed between selenium exposure and ALT lies at selenium intake levels slightly higher than the recommended dietary allowance which is generally set around 45 µg/day but also ranges between 26 and 70 µg/selenium/day according to different authorities [11,72,73,74]. Such intake is also below the current upper intake levels, although these appear to need a reassessment [1]. To conclude, the multiple mechanisms in which selenium may exert its toxicological effects certainly need further investigations. Future research should focus on the role of the different selenium species on oxidoreduction status [70,75,76], protein integrity [77] as well as the microbiome, a research area of growing interest due to its relation with several chronic diseases [78,79] and its possible susceptibility to the adverse effects of selenium [80].

## 5. Conclusions

Our results suggest the existence of a positive relation between selenium exposure and ALT levels. This association appears to be enhanced in relation to selected selenium organic species like Se-Cys and Se-GPX, and to an inorganic selenium form, selenite. Our findings provide new evidence on the possible adverse metabolic effects of selenium, even at concentrations below the upper intake levels set by international agencies.

## Figures and Tables

**Table 1:** Characteristics of the study population and mean levels of urinary selenium (*n* = 137), dietary selenium intake (*n* = 137), and serum selenium (*n* = 104).

Characteristics	All					Men					Women				
	N	%	Urinary Se (µg/L)	Dietary Se (µg/day)	Serum Se (µg/day)	N	%	Urinary Se (µg/L)	Dietary Se (µg/day)	Serum Se (µg/day)	N	%	Urinary Se (µg/L)	Dietary Se (µg/day)	Serum Se (µg/day)
Overall	137	100	26.77	84.09	117.44	62	45.3	29.00	89.97	119.22	75	54.7	24.92	79.23	115.80
Age															
<50 years	80	58.4	27.23	86.13	116.75	39	62.9	30.17	90.94	119.03	41	54.7	24.44	81.55	114.47
≥50 years	57	41.6	26.12	81.24	118.39	23	37.1	27.04	88.32	119.50	34	45.3	25.50	76.44	117.46
Body Mass Index															
<25 kg/m^2^	74	54.0	25.59	82.21	116.50	32	51.6	28.47	91.10	116.92	42	56.0	23.39	75.44	116.13
≥25 kg/m^2^–<30 kg/m^2^	50	36.5	28.58	84.22	119.85	27	43.6	29.49	87.04	122.14	23	30.7	27.52	80.92	117.06
≥30 kg/m^2^	13	9.5	26.52	94.31	112.00	3	4.8	30.40	104.27	117.00	10	13.3	25.36	91.32	110.33
Smoking habits															
Never	101	73.7	26.10	83.95	117.49	45	72.6	28.77	88.60	118.94	56	74.7	23.95	80.21	116.24
Former	36	26.3	28.66	84.50	117.32	17	27.4	29.64	93.60	119.87	19	25.3	27.79	76.36	114.38
Selenium supplement users															
No	94	68.6	25.06	87.56	117.27	46	74.2	29.80	94.65	119.03	48	64.0	22.46	80.76	115.42
Yes	23	16.8	29.08	80.14	118.69	6	9.7	27.75	72.37	122.00	17	22.7	29.55	82.88	117.22
Former	20	14.6	27.48	72.34	117.23	10	16.1	26.11	78.97	118.67	10	13.3	28.85	65.71	116.00
Marital status															
Married/unmarried partner	97	70.8	26.78	83.10	116.68	44	71.0	29.72	87.69	117.77	53	70.7	24.34	79.28	115.71
Single	26	19.0	27.65	87.72	119.25	12	19.4	28.80	104.28	121.80	14	18.7	26.66	73.52	116.70
Separated/divorced	14	10.2	25.07	84.26	119.00	6	9.6	24.20	78.05	123.17	8	10.7	25.73	88.91	114.83
Educational level															
Elementary school	2	1.5	37.26	146.96	131.50	2	3.2	37.26	146.96	131.50	-	-	-	-	-
Middle school	20	14.6	25.99	84.79	120.07	8	12.9	29.46	80.08	126.14	12	16.0	23.67	87.92	114.75
High school	66	48.2	23.87	82.73	116.20	28	45.2	24.98	90.92	114.20	38	50.7	23.05	76.70	117.93
College or more	49	35.8	30.57	83.07	117.43	24	38.7	32.87	87.40	122.50	25	33.3	28.37	78.92	112.65
Occupation															
Managers	9	6.6	21.30	84.83	118.58	6	9.7	21.42	80.45	117.51	3	4.0	21.04	93.60	120.00
Professionals	26	19.0	30.16	91.07	119.21	12	19.4	33.81	104.05	130.11	14	18.7	27.03	79.93	109.40
Technicians/associate professionals	21	15.3	25.96	82.74	115.00	11	17.7	23.41	89.03	116.89	10	13.3	28.75	75.82	112.57
Clerical support workers	43	31.4	25.78	79.34	115.48	12	19.4	32.10	82.18	104.50	31	41.3	22.99	78.24	119.00
Service and sales workers	11	8.0	26.65	71.98	111.33	2	3.2	22.71	81.07	108.00	9	12.0	27.52	69.96	113.00
Craft and related trade workers	10	7.3	22.16	80.73	114.60	8	12.9	24.80	82.40	114.63	2	2.7	11.61	74.09	114.50
Plant and machine operators	11	8.0	35.63	94.40	122.63	8	12.9	36.22	100.51	126.29	3	4.0	34.05	78.12	97.00
Elementary occupations	6	4.4	21.92	100.44	134.60	3	4.8	25.78	85.25	138.33	3	4.0	18.07	115.63	129.00

**Table 2:** Median and interquartile range (IQR) of alanine aminotransferase, dietary, urinary (*n* = 137), and total serum selenium (*n* = 104) in our study population.

	All (*n* = 137)		Men (*n* = 62)		Women (*n* = 75)	
	Median	IQR	Median	IQR	Median	IQR
ALT (U/L)	27	22–35	30	26–43	24	20–29
Dietary Se intake (µg/day)	78.74	62.62–101.48	88.37	69.77–108.28	71.06	54.77–91.68
Urinary Se concentration (µg/L)	22.02	14.64–37.15	24.21	16.72–39.20	21.30	13.30–34.66
Total serum Se concentration (µg/L) ^1^	116.50	106.00–128.00	118.00	109.00–132.00	115.00	105.00–125.00

^1^ Total serum Se concentration data were available for 104 subjects (50 men and 54 women).

**Table 3:** Median and interquartile range (IQR) of selenium species in serum in our study population (*n* = 104).

	All (*n* = 104)		Men (*n* = 50)		Women (*n* = 54)	
	Median	IQR	Median	IQR	Median	IQR
Organic Se (µg/L)	102.24	84.53–113.86	100.56	79.28–117.28	102.58	87.24–112.13
SELENOP (µg/L)	84.81	64.23–104.05	79.44	59.88–105.03	86.02	72.48–101.65
Se-GPX (µg/L)	5.46	2.12–8.88	3.72	1.47–9.03	5.74	2.45–8.16
Se-TXNRD (µg/L)	0.40	0.03–1.51	0.46	0.03–1.66	0.12	0.03–1.31
Se-Met (µg/L)	2.74	1.41–5.23	2.81	1.63–5.10	2.55	1.23–5.67
Se-Cys (µg/L)	1.91	0.45–3.61	1.93	0.42–4.06	1.91	0.49–3.43
Inorganic Se (µg/L)	9.67	3.73–22.94	10.02	3.66–28.70	8.74	3.74–19.28
Se (IV) (µg/L)	4.37	1.57–13.83	4.38	1.21–19.09	4.37	1.77–11.95
Se (VI) (µg/L)	3.30	1.01–8.17	3.85	0.97–9.46	3.07	1.04–7.61
Se-HSA (µg/L)	1.12	0.03–3.06	1.06	0.03–4.02	1.24	0.03–2.85

Abbreviations: SELENOP, selenoprotein P; Se-GPX, glutathione peroxidase-bound selenium; Se-TXNRD, thioredoxin reductase-bound selenium; Se-Met, selenomethionine; Se-Cys, selenocysteine; Se (IV), selenite; Se (VI), selenate; Se-HSA, human serum albumin-bound selenium.

**Table 4:** Linear regression analyses of alanine aminotransferase (ALT) versus urinary selenium (Se) concentration, dietary Se intake, total serum Se concentration, organic inorganic, and human serum albumin-bound selenium biomarkers as independent variables. We reported two statistical models: credo and adjusted by age, sex, body mass index, cotinine levels, and intake of alcohol. Values are beta coefficients (β) with 95% confidence intervals (CI).

Selenium Biomarkers	Crude		Adjusted	
	β	(95% CI)	β	(95% CI)
Urinary Se concentration (µg/L)	0.14	(0.03, 0.26)	0.11	(0.003, 0.21)
Dietary Se intake (µg/day)	0.07	(0.004, 0.13)	0.03	(−0.02, 0.09)
Total serum Se concentration (µg/L) ^1^	−0.04	(−0.16, 0.09)	−0.04	(−0.16, 0.08)
Organic Se (µg/L)	−0.02	(−0.09, 0.06)	0.01	(−0.06, 0.08)
*SELENOP* (µg/L)	−0.02	(−0.09, 0.05)	0.01	(−0.06, 0.07)
*Se-GPX* (µg/L)	0.06	(−0.33, 0.45)	0.09	(−0.29, 0.47)
*Se-TXNRD* (µg/L) ^2^	0.17	(−1.22, 1.57)	−0.24	(−1.55, 1.07)
*Se-Met* (µg/L) ^2^	0.03	(−0.46, 0.51)	0.04	(−0.41, 0.49)
*Se-Cys* (µg/L) ^2^	0.30	(−0.57, 1.18)	0.27	(−0.55, 1.10)
Inorganic Se (µg/L)	−0.001	(−0.10, 0.10)	−0.02	(−0.11, 0.07)
*Se (IV)* (µg/L) ^2^	0.03	(−0.12, 0.19)	−0.002	(−0.15, 0.14)
*Se (VI)* (µg/L) ^2^	−0.14	(−0.37, 0.10)	−0.14	(−0.36, 0.08)
Se-HSA (µg/L) ^2^	−0.54	(−1.13, 0.06)	−0.56	(−1.11, −0.01)

^1^ Regression estimate calculated upon variable winsorized at 1st percentile. ^2^ Regression estimate calculated upon variable winsorized at 99th percentile. Abbreviations: SELENOP, selenoprotein P; Se-GPX, glutathione peroxidase-bound selenium; Se-TXNRD, thioredoxin reductase-bound selenium; Se-Met, selenomethionine; Se-Cys, selenocysteine; Se (IV), selenite; Se (VI), selenate; Se-HSA, human serum albumin-bound selenium.

## Data Availability

The data presented in this study may be available on reasonable request from the corresponding author. The data are not publicly available due to privacy and legal restrictions.

## Supplementary Materials

The following are available online at https://www.mdpi.com/article/10.3390/antiox10101516/s1, Table S1: Sex-specific association of alanine aminotransferase (ALT) with urinary selenium (Se) concentration and dietary selenium (Se) intake (*n* = 62), and with total serum Se and Se species concentrations (*n* = 50) in crude and adjusted linear regression analysis. Figure S1: Spline regression analysis of the associations between urinary, dietary, and serum selenium in the entire study population (*n* = 137). The solid line represents a multivariable estimate (adjusted by age, sex, body mass index, cotinine levels, and intake of alcohol) and the shaded area 95% confidence interval. Figure S2: Spline regression analysis of urinary, dietary, and total serum selenium (Se) levels versus ALT in men. The solid line represents multivariable analysis estimates (adjusted by age, body mass index, cotinine levels, and intake of alcohol) and shaded area the 95% confidence interval. Figure S3: Spline regression analysis of urinary, dietary, and total serum selenium (Se) levels versus ALT in women. The solid line represents multivariable analysis estimates (adjusted by age, body mass index, cotinine levels, and intake of alcohol) and shaded area the 95% confidence interval. Figure S4: Spline regression analysis of total organic serum selenium (Se) levels and organic species versus ALT in men. The solid line represents multivariable analysis estimates (adjusted by age, body mass index, cotinine levels, and intake of alcohol) and shaded area the 95% confidence interval. Figure S5: Spline regression analysis of total organic serum selenium (Se) levels and organic species versus ALT in women. The solid line represents multivariable analysis (adjusted by age, body mass index, cotinine levels, and intake of alcohol) and shaded area the 95% confidence interval. Figure S6: Spline regression analysis of total inorganic serum selenium (Se) levels, inorganic species, and Human Serum Albumin-bound selenium (Se-HSA) versus ALT in men. The solid line represents multivariable analysis estimates (adjusted by age, body mass index, cotinine levels, and intake of alcohol) and shaded area the 95% confidence interval. Figure S7: Spline regression analysis of total inorganic serum selenium (Se) levels, inorganic species, and Human Serum Albumin-bound selenium (Se-HSA) versus ALT in women. The solid line represents multivariable analysis estimates (adjusted by age, body mass index, cotinine levels, and intake of alcohol) and shaded area the 95% confidence interval.
