# Supplementary material for: Association of Urinary and Dietary Selenium and of Serum Selenium Species with Serum Alanine Aminotransferase in a Healthy Italian Population
Source: Antioxidants (Basel). 2021 Sep 24;10(10):1516. doi: 10.3390/antiox10101516 (PMC8532767; doi:10.3390/antiox10101516)

antioxidants

MDPI

Table S1. Sex specific association of alanine aminotransferase (ALT) with urinary selenium (Se) concentration and dietary selenium (Se) intake (n=62), and with total serum Se and Se species concentrations (n=50) in crude and adjusted linear regression analysis.

Men	Crude		Adjusted [1]	
	β	(95% CI)	β	(95% CI)
Urinary Se concentration (µg/L)	0.21	(0.03, 0.39)	0.20	(0.03, 0.37)
Dietary Se intake (µg/day)	0.11	(0.003, 0.22)	0.11	(0.02, 0.21)
Total serum Se concentration (µg/L) [2]	-0.09	(-0.27, 0.09)	-0.06	(-0.25, 0.13)
Total organic Se (µg/L)	0.01	(-0.10, 0.11)	-0.01	(-0.11, 0.10)
Se-SELENOP (µg/L)	0.01	(-0.08, 0.11)	-0.01	(-0.11, 0.09)
Se-GPX (µg/L)	-0.03	(-0.59, 0.54)	0.12	(-0.51, 0.74)
Se-TXNRD (µg/L) [3]	-0.43	(-2.43, 1.58)	-0.14	(-2.10, 1.81)
Se-Met (µg/L)	-0.13	(-0.99, 0.74)	-0.08	(-0.92, 0.76)
Se-Cys (µg/L)	0.001	(-1.49, 1.49)	0.29	(-1.20, 1.76)
Total inorganic Se (µg/L)	-0.06	(-0.18, 0.06)	-0.03	(-0.15, 0.10)
Se (IV) (µg/L) [3]	-0.07	(-0.27, 0.13)	-0.02	(-0.21, 0.18)
Se (VI) (µg/L) [3]	-0.27	(-0.58, 0.05)	-0.19	(-0.51, 0.12)
Se-HSA (µg/L)	-0.74	(-1.60, 0.12)	-0.57	(-1.48, 0.35)
**Women**	**Crude**		**Adjusted [1]**	
	β	(95% CI)	β	(95% CI)
Urinary Se concentration (µg/L)	0.02	(-0.11, 0.14)	0.04	(-0.08, 0.16)
Dietary Se intake (µg/day)	-0.004	(-0.07, 0.06)	-0.02	(-0.08, 0.04)
Total serum Se concentration (µg/L)	-0.03	(-0.19, 0.13)	0.01	(-0.15, 0.17)
Organic Se (µg/L)	-0.03	(-0.14, 0.08)	-0.01	(-0.12, 0.09)
Se- SELENOP (µg/L)	-0.06	(-0.16, 0.05)	-0.03	(-0.13, 0.07)
Se-GPX (µg/L)	0.17	(-0.31, 0.65)	0.17	(-0.29, 0.62)
Se- TXNRD (µg/L)	0.19	(-1.58, 1.97)	-0.22	(-1.98, 1.53)
Se-Met (µg/L) [3]	0.22	(-0.25, 0.70)	0.12	(-0.35, 0.59)
Se-Cys (µg/L) [3]	0.46	(-0.44, 1.36)	0.21	(-0.67, 1.11)
Inorganic Se (µg/L)	0.07	(-0.11, 0.24)	0.07	(-0.10, 0.23)
Se (IV) (µg/L)	0.15	(-0.11, 0.41)	0.13	(-0.12, 0.38)
Se (VI) (µg/L)	-0.002	(-0.33, 0.32)	0.04	(-0.27, 0.35)
Se-HSA (µg/L) [3]	-0.42	(-1.14, 0.31)	-0.28	(-0.98, 0.43)

[1] Adjusted in a multivariable model for age, sex, body mass index (BMI), cotinine levels, and alcohol intake along with their 95% confidence interval (CI); [2] Regression estimates calculated upon variable winsorized at 1st percentile; [3] Regression estimates calculated upon variable winsorized at 99th percentile.

Figure S1. Spline regression analysis of the associations between urinary, dietary and serum selenium in the entire study population (n=137). Solid line represents multivariable estimate (adjusted by age, sex, body mass index, cotinine levels, and intake of alcohol) and the shaded area 95% confidence interval.

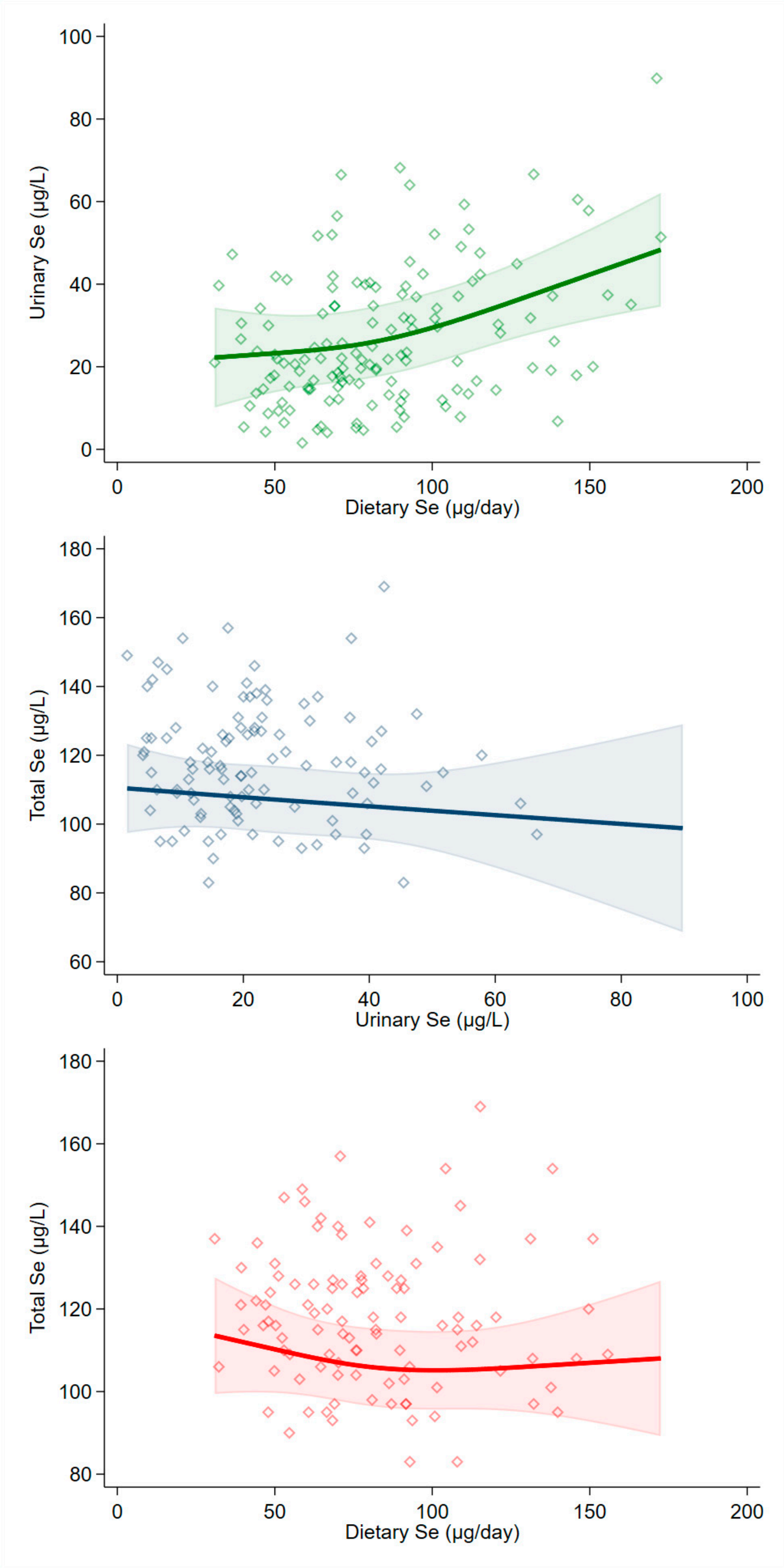

Figure S2. Spline regression analysis of urinary, dietary, and total serum selenium (Se) levels versus ALT in men. Solid line represents multivariable analysis estimates (adjusted by age, body mass index, cotinine levels, and intake of alcohol) and shaded area the 95% confidence interval.

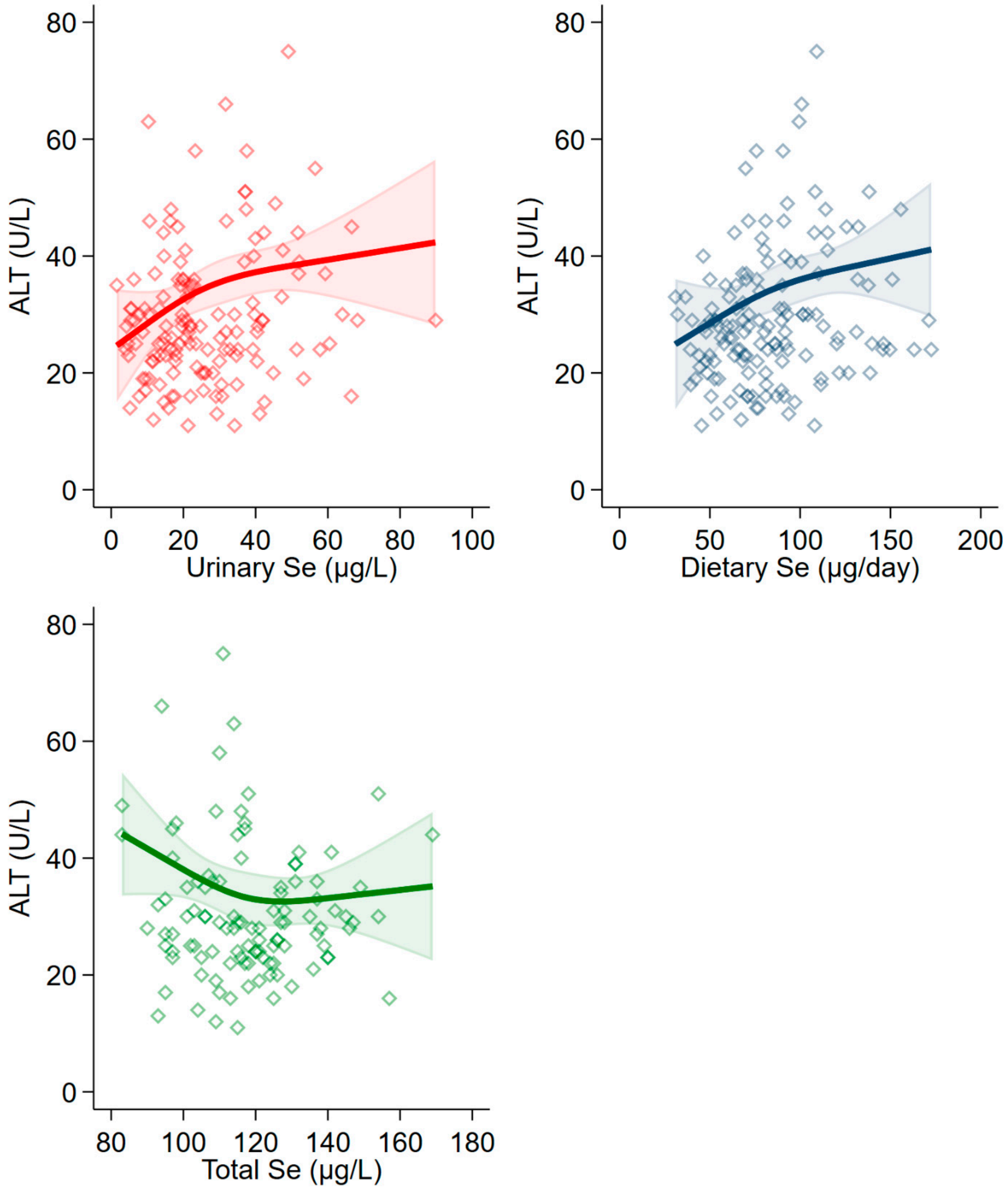

Figure S3. Spline regression analysis of urinary, dietary, and total serum selenium (Se) levels versus ALT in women. Solid line represents multivariable analysis estimates (adjusted by age, body mass index, cotinine levels, and intake of alcohol) and shaded area the 95% confidence interval.

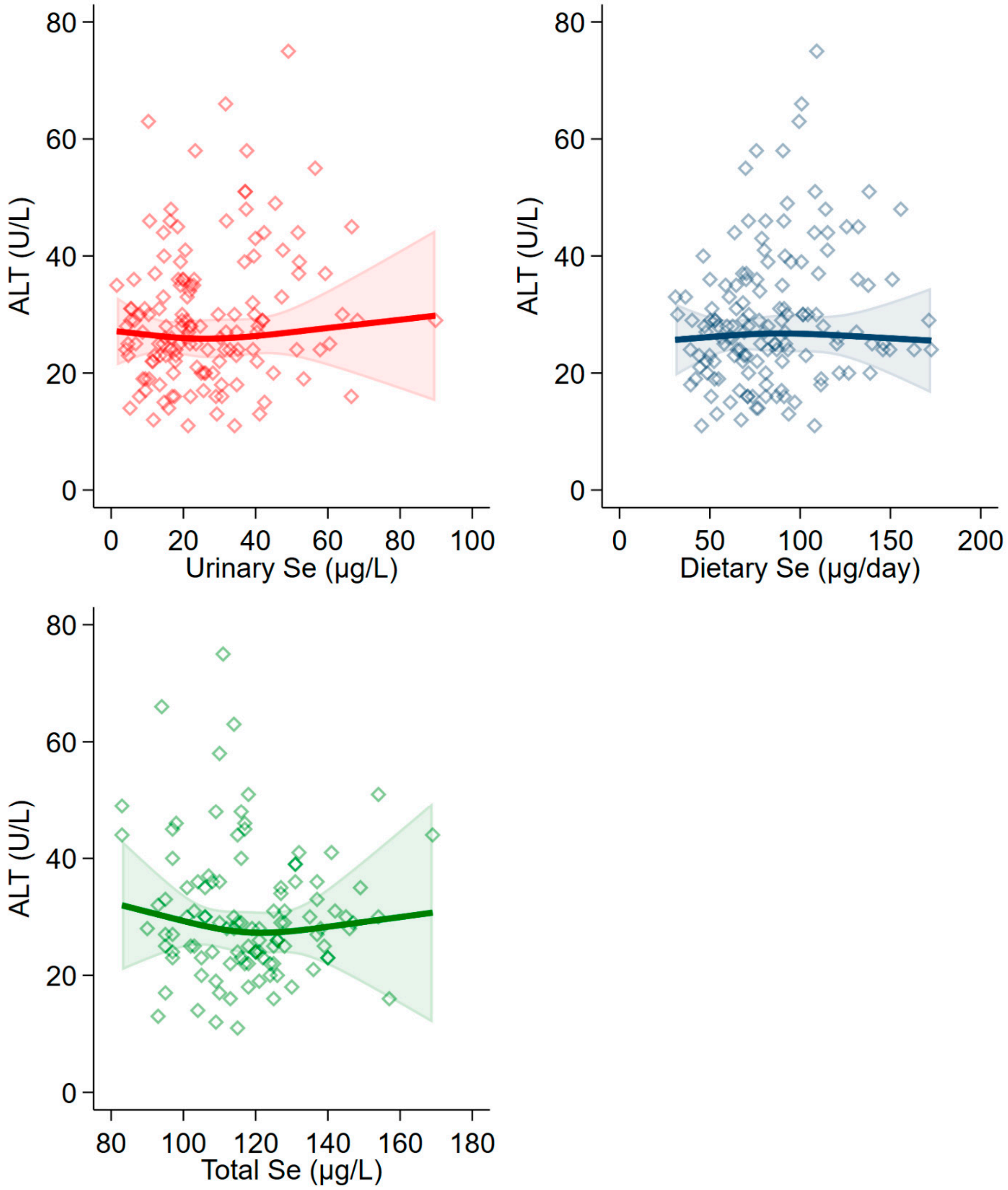

Figure S4. Spline regression analysis of total organic serum selenium (Se) levels and organic species versus ALT in men. Solid line represents multivariable analysis estimates (adjusted by age, body mass index, cotinine levels, and intake of alcohol) and shaded area the 95% confidence interval.

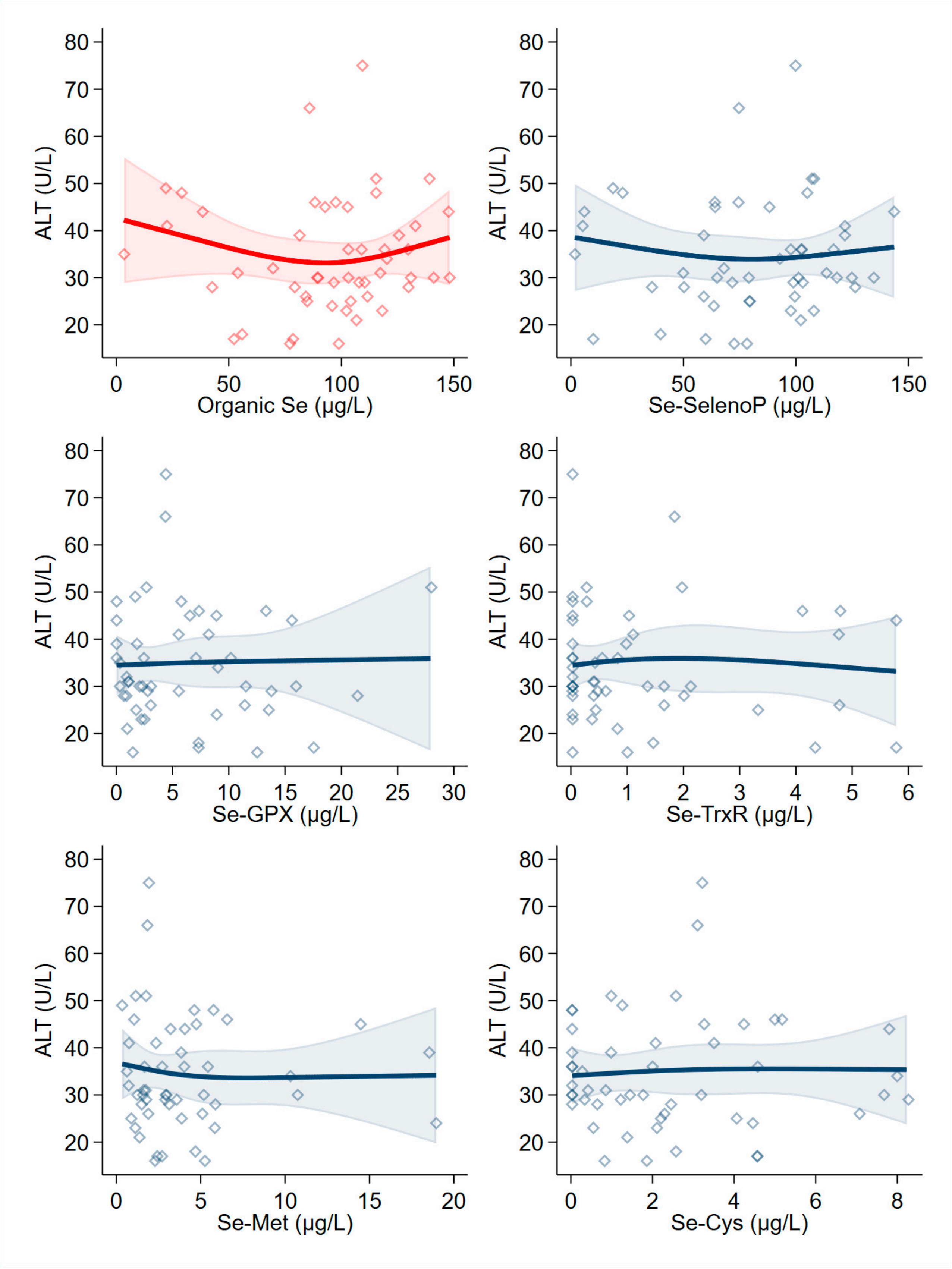

Figure S5. Spline regression analysis of total organic serum selenium (Se) levels and organic species versus ALT in women. Solid line represents multivariable analysis estimates (adjusted by age, body mass index, cotinine levels, and intake of alcohol) and shaded area the 95% confidence interval.

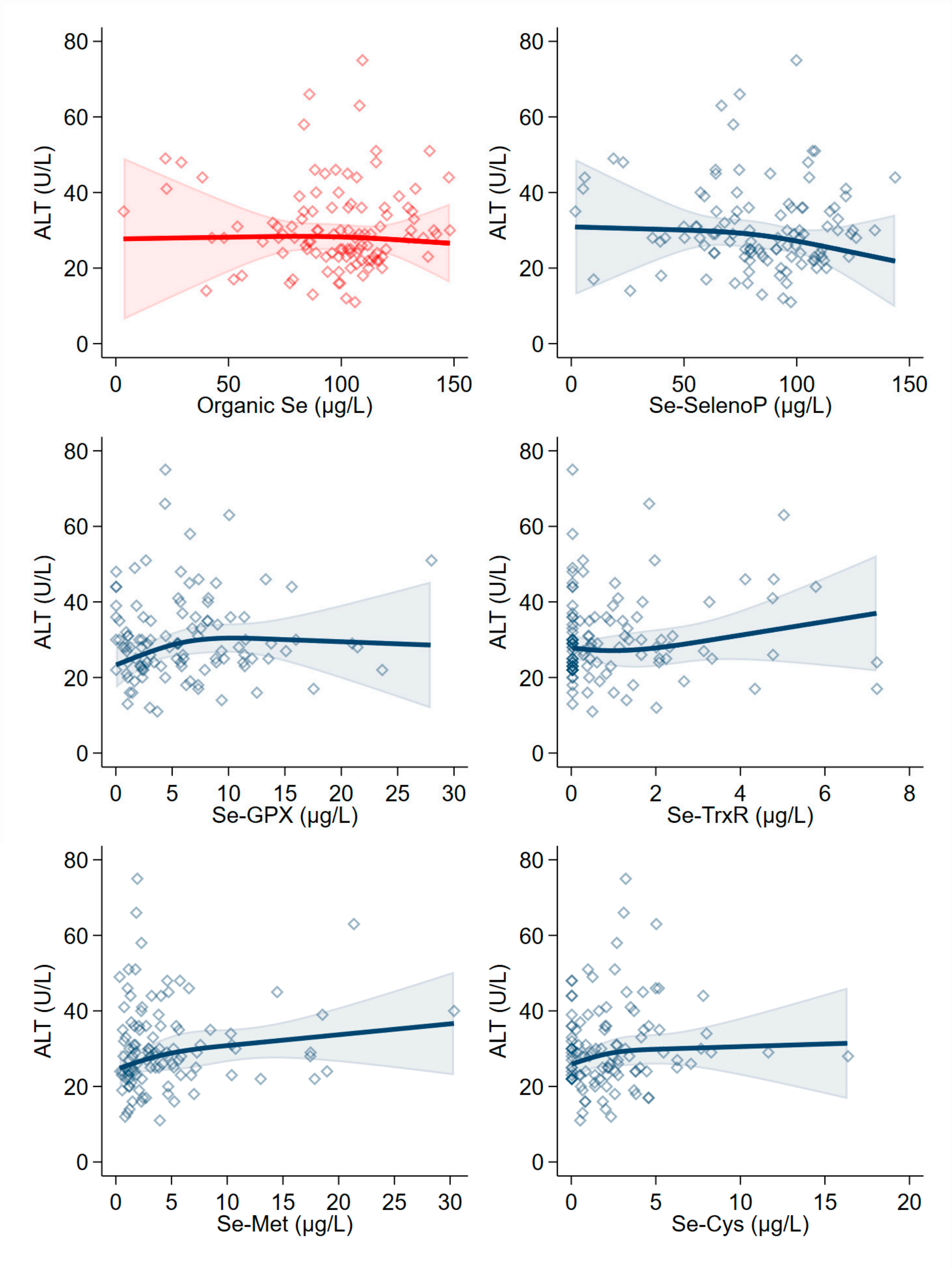

Figure S6. Spline regression analysis of total inorganic serum selenium (Se) levels, inorganic species, and Human Serum Albumin-bound selenium (Se-HSA) versus ALT in men. Solid line represents multivariable analysis estimates (adjusted by age, body mass index, cotinine levels, and intake of alcohol) and shaded area the 95% confidence interval.

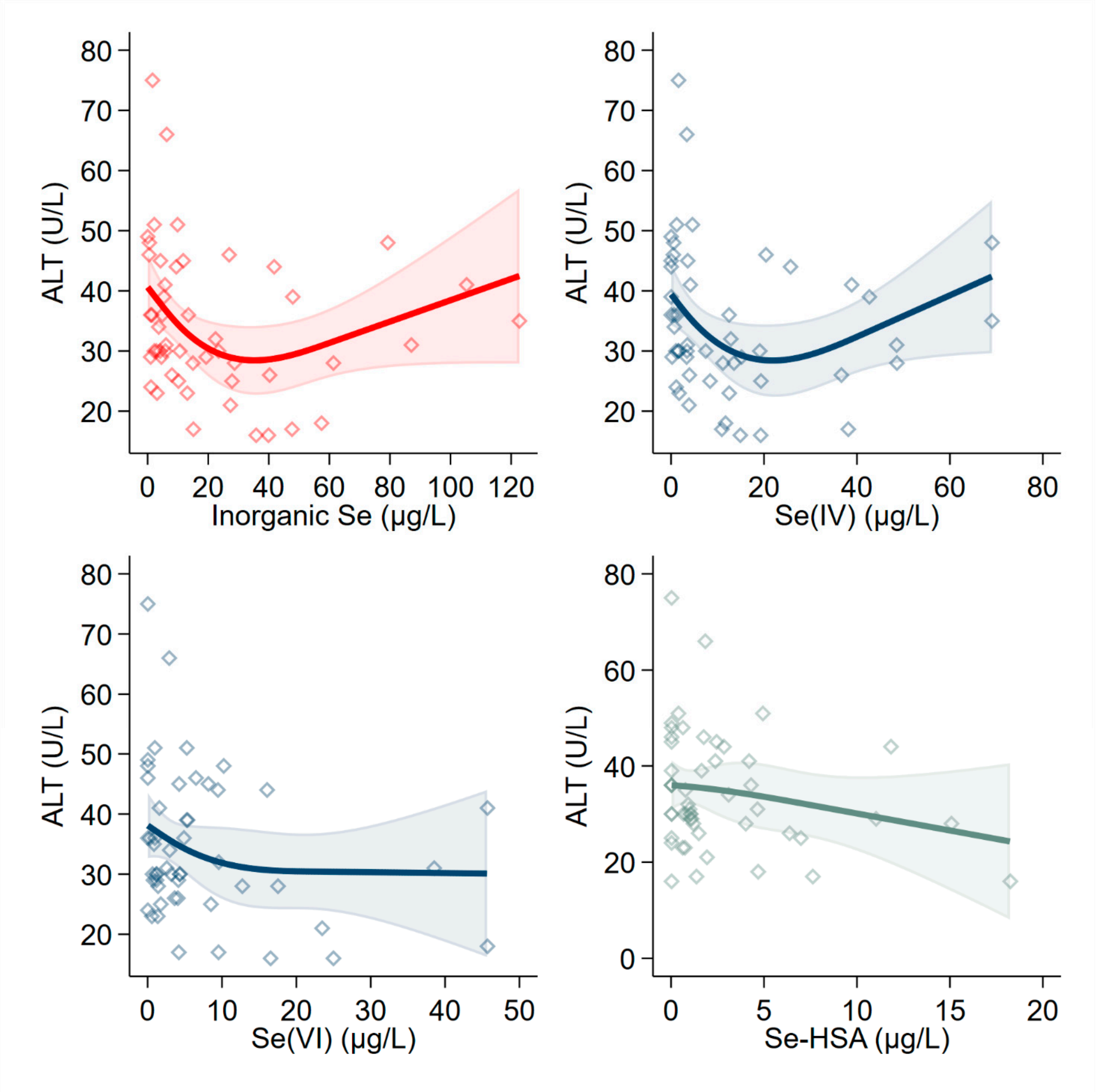

Figure S7. Spline regression analysis of total inorganic serum selenium (Se) levels, inorganic species, and Human Serum Albumin-bound selenium (Se-HSA) versus ALT in women. Solid line represents multivariable analysis estimates (adjusted by age, body mass index, cotinine levels, and intake of alcohol) and shaded area the 95% confidence interval.

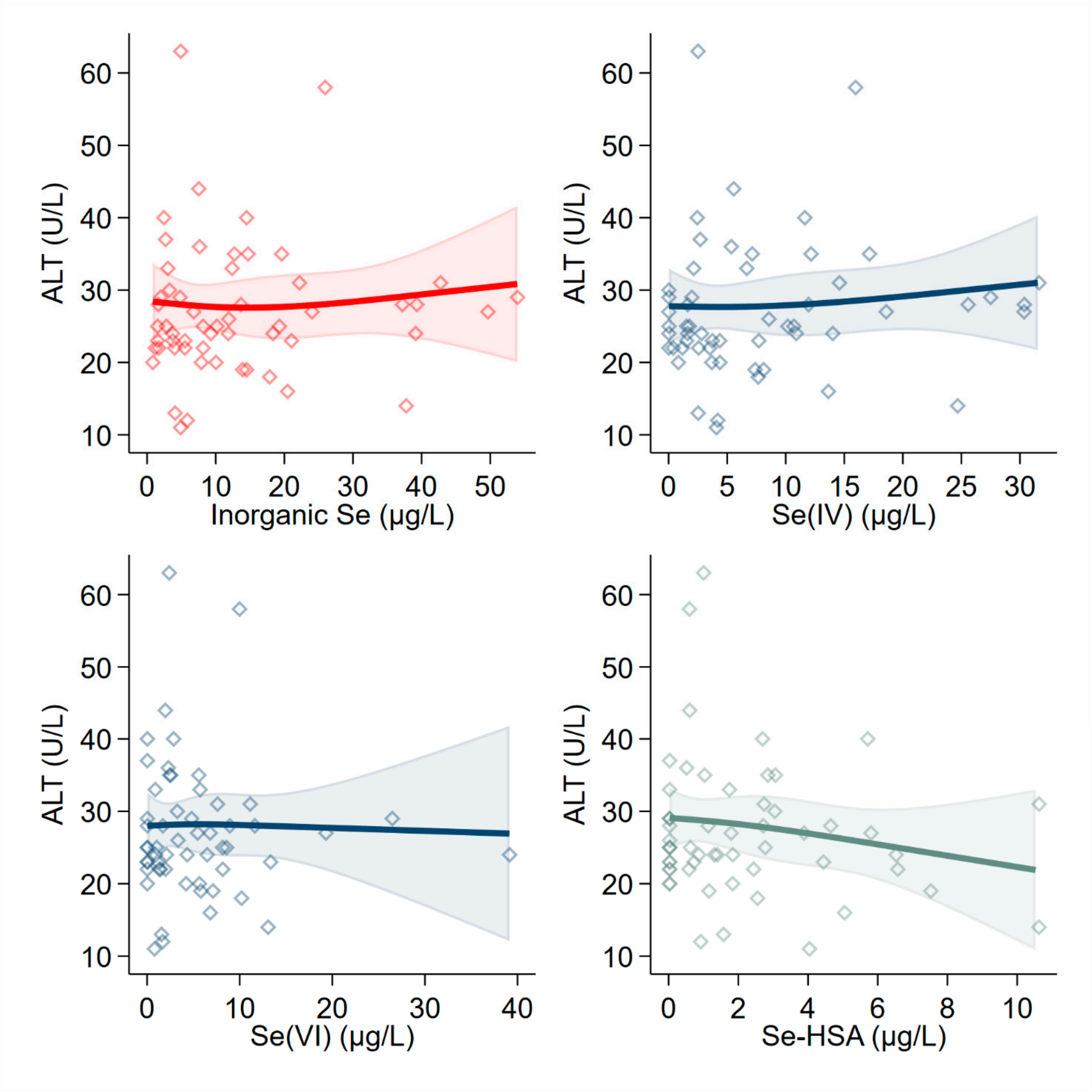

Supplement: Supplementary file 1 [file antioxidants-10-01516-s001.zip › antioxidants-1369128-supplementary.pdf]
